# Sudden seizure during cesarean section: A case report

**DOI:** 10.1097/MD.0000000000013785

**Published:** 2018-12-28

**Authors:** Wenqin Zhou, Qing Zhu

**Affiliations:** a Department of Anesthesiology, West China Second University Hospital, Sichuan University; b Key Laboratory of Birth Defects and Related Diseases of Women and Children, Ministry of Education (Sichuan University), Chengdu, China.

**Keywords:** cesarean delivery, seizure, spinal anesthesia

## Abstract

**Rationale:**

A first seizure can range from a fleeting subjective experience or a twitch (myoclonic jerk) through to a tonic-clonic convulsion. A first seizure occurred in a parturient during cesarean section is very rare. We describe the case of a parturient who suffered seizure when the fetus was delivered.

**Patient concerns:**

Our patient is a 31-year-old parturient with a first seizure during cesarean section.

**Diagnoses:**

Seizure.

**Interventions:**

The patient received supportive therapy to maintain oxygen supply and propofol was administered to terminate seizure during cesarean section.

**Outcomes:**

after therapy, the patient regained full consciousness and normal spontaneous respiration. She had no recall of the seizure attack. The post-operative recovery was uneventful.

**Lessons:**

Symptoms during seizure are very important to diagnose. It will be both harmful to mother and the fetus, when the pregnant woman suffers seizure during pregnancy or cesarean delivery. In this situation, supportive therapy need to be immediately initiate and propofol may be the most suitable drug to terminate seizure during cesarean delivery.

## 1 Introduction

A seizure is a transient disturbance of cerebral function caused by abnormal, paroxysmal, hypersynchronous electrical neuronal activity in the cerebral cortex. The etiology of seizures covers a wide spectrum of diseases and disorders. A first seizure can range from a fleeting subjective experience or a twitch (myoclonic jerk) through to a tonic-clonic convulsion.^[1]^ It is important to distinguish seizures from events that can mimic epilepsy and to recognize that risk factors that temporarily lower seizure threshold in an otherwise normal brain.^[2]^ Epilepsy is defined as more than one seizure and proper history taking is the most important tool to diagnose. Important information includes: previous history of seizures, stroke, brain tumor or trauma, past obstetrical history, and precipitating events (medications, alcohol, and illicit drugs).^[3]^ Electrocardiogram (EEG) is recommended by the American Academy of Neurology and the American Society of Epilepsy as part of the initial neurodiagnostic evaluation.^[4]^ A first seizure may be provoked or unprovoked. It is generally accepted that, pregnancy and labor, and the post-partum, or post-natal period (after birth) can be risk factors for seizure.

There is no literature about a parturient who suffers seizure during cesarean section. This article presented a case of a first seizure in a parturient when the fetus was delivered.

## 2 Case report

A 31-year-old pregnant woman presented to the department of emergency with painless vaginal bleeding for 4 hours, at the gestational age of 33 weeks. The patient's previous medical history was unremarkable, except that this current pregnancy was complicated with placenta previa. The parturient was scheduled for elective cesarean delivery, after receiving magnesium sulfate for uterine relaxaion and dexamethasone for promoting fetal lung maturation for consecutive 13 days.

Arriving in the operating room, the patient was in great anxiety, with a heart rate of 122 beats/min, noninvasive blood pressure of 110/68 mmHg, and pulse oxygen saturation of 100%. Spinal anesthesia was performed in lateral recumbent position at the L3 to L4 interspace, with 2.5 mL of 0.5% isobaric bupivacaine. The sensory blockade reached the level of T4. When obstetrician made an incision in the lower segment of the uterus to deliver the fetus, a sudden seizure occurred and the parturient lost her consciousness. There were accompanying rhythmic twitching of upper limbs, trunk, and face. The eyes were deviated to the upside and trismus developed with increased oropharyngeal secretions. At the meantime, the blood pressure was 64/33 mmHg and heart rate was 59 beats/min, while the pulse oxygen saturation could not be detected by the SpO_2_ sensor.

Jaw thrust and mask ventilation with 100% oxygen were immediately applied. Then, 100 mg propofol was given to terminate the seizure, and 6 mg ephedrine was administrated to stabilize the hemodynamics. Meanwhile, the newborn was delivered, and the Apgar scores at 1-, 5-, and 10- minute were 10, 10, and 10, respectively. One minute later, convulsion resolved. Since the patient was still in apnea, an oropharyngeal airway was inserted to keep the airway open and facilitate mask ventilation. At this moment, pulse oxygen saturation was 100%, and blood pressure and heart rate were 98/45 mmHg and 100 beats/min, respectively. The artery blood gas analysis was performed which showed that: pH 7.402, PO_2_ 82 mmHg, PCO_2_ 28.2 mmHg, BE-7, HCO_3_^−^ 17.5 mmol/L, K^+^ 3.7 mmol/L, Na^+^ 135 mmol/L, HCT 7.5 g/L, Glu 6.3 mmol/L. The body temperature was 36.2 °C. Five more minutes, the patient regained full consciousness and normal spontaneous respiration. She had no recall of the seizure attack. The postoperative recovery was uneventful. Furthermore, the cranial computed tomography scan revealed no abnormalities.

## 3 Discussion

This is the first case of a parturient who suffers seizure during cesarean section. The seizure suddenly occurred during surgery, so we couldn’t perform EEG test or routine neurological examination immediately. We took a computed tomography scan but no positive findings were presented. In summary, the parturient had no risk factors in previous medical history and never had seizure before or in the following days in hospital, we consider this issue should be a first seizure in adults. A first seizure may be provoked or unprovoked. It is generally accepted that, pregnancy and labor, and the post-partum, or post-natal period (after birth) can be risk factors for seizure, especially if there are certain complications like eclampsia. Eclampsia is defined as the new onset of seizures and/or unexplained coma during pregnancy or postpartum in patients with signs and symptoms of preeclampsia, but without a pre-existing neurologic disorder.^[5]^ Eclamptic seizures typically occur suddenly. The differential diagnosis for seizures is broad, however, all seizures in the peripartum period should be considered eclampsia until proven otherwise, and treatment should be initiated immediately. Hypertension is considered the hallmark for the diagnosis of eclampsia, and in this case, the absence of hypertension and proteinuria helps to distinguish this condition from eclampsia.

Under spinal or epidural anesthesia, circulation is greatly influenced by the blockade of the sympathetic tone. What's more important is that when obstetrician is delivering the baby from uterus, strongly peritoneal traction may further aggravate the increase in parasympathetic activity. The hypotension and bradycardia in this case, supports the assumption. Severe hypotension may lead to transient cerebral hypoxia, which is likely to cause seizure. Once the seizure occurs during the cesarean delivery, supportive therapy such as oxygen, mechanical ventilation should be instituted as needed. The aim is to terminate the seizure as rapidly as possible. There are 4 main categories of drugs that are used to treat status epilepticus: benzodiazepines, phenytoin, barbiturates, and propofol. Both maternal and fetal safety should be considered when choosing the medication to terminate seizures in parturient. Therefore, potent sedatives such as benzodiazepines and barbiturates should be used with caution to avoid the risk respiratory depression and aspiration, although they are effective in seizure control.^[6]^ Propofol is a rapid onset and short-lasting intravenous anesthetic. When given as an intravenous bolus, propofol rapidly crosses the placenta and results in an umbilical vein-to-maternal vein (UV/MV) ratio of approximately 0.7.^[7]^ It has been well demonstrated that low doses of propofol (<2 mg/kg) have no effect on fetal Apgar and neurobehavioral scores.^[8,9]^ However, propofol results in a greater incidence of maternal hypotension, especially with patients with increased parasympathetic activity, as occurred in this current case. We should pay enough attention to this. Ongoing anti-epileptic medications are not typically recommended after a first seizure except in those with structural lesions in the brain. They are generally recommended after the occurrence of second one.

## 4 Conclusion

A seizure is a clinical manifestation of presumed or proved abnormal electrical activity in the brain. A first seizure can be provoked or unprovoked. Epilepsy is defined as more than one seizure. Symptoms during seizure are very important to diagnose. It will be both harmful to mother and the fetus, when the pregnant woman suffers seizure during pregnancy or cesarean delivery. In this situation, supportive therapy need to be initiate immediately and propofol may be the most suitable drug to terminate seizure during cesarean delivery.

## Author contributions

Wenqin Zhou collected the case and drafted the manuscript.

Qing Zhu edited the manuscript and final approval of manuscript.

**Writing – original draft:** Wenqin Zhou.

**Writing – review & editing:** Qing Zhu.
